# An Intra-Hospital Spread of Colistin-Resistant *K. pneumoniae* Isolates—Epidemiological, Clinical, and Genetic Analysis

**DOI:** 10.3390/medicina60030511

**Published:** 2024-03-21

**Authors:** Georgi Popivanov, Rumyana Markovska, Ivanka Gergova, Marina Konaktchieva, Roberto Cirocchi, Kirien Kjossev, Ventsislav Mutafchiyski

**Affiliations:** 1 Department of Surgery, Military Medical Academy, 1606 Sofia, Bulgaria; kirienkt@gmail.com (K.K.); ventzimm@gmail.com (V.M.); 2 Department of Medical Microbiology, Medical Faculty, Medical University, 1431 Sofia, Bulgaria; markovska73@abv.bg; 3 Department of Microbiology and Virology, Military Medical Academy, 1606 Sofia, Bulgaria; gergova.ivana7@gmail.com; 4 Department of Gastroenterology and Hepatology, Military Medical Academy, 1606 Sofia, Bulgaria; marina.konaktchieva@yahoo.com; 5 Department of Surgical Science, University of Perugia, 06100 Perugia, Italy; roberto.cirocchi@unipg.it

**Keywords:** outbreak, colistin-resistant *K. pneumoniae*, *mgrB*, plasmid-mediated resistance, mortality

## Abstract

*Background and Objective: Klebsiella pneumoniae* appears to be a significant problem due to its ability to accumulate antibiotic-resistance genes. After 2013, alarming colistin resistance rates among carbapenem-resistant *K. pneumoniae* have been reported in the Balkans. The study aims to perform an epidemiological, clinical, and genetic analysis of a local outbreak of COLr CR-Kp. *Material and Methods:* All carbapenem-resistant and colistin-resistant *K. pneumoniae* isolates observed among patients in the ICU unit of Military Medical Academy, Sofia, from 1 January to 31 October 2023, were included. The results were analyzed according to the EUCAST criteria. All isolates were screened for *bla*VIM, *bla*IMP, *bla*KPC, *bla*NDM, and *bla*OXA-48. Genetic similarity was determined using the Dice coefficient as a similarity measure and the unweighted pair group method with arithmetic mean (UPGMA). *mgrB* genes and plasmid-mediated colistin resistance determinants (*mcr-1*, *mcr-2*, *mcr-3*, *mcr-4*, and *mcr-5*) were investigated. *Results:* There was a total of 379 multidrug-resistant *K. pneumoniae* isolates, 88% of which were carbapenem-resistant. Of these, there were nine (2.7%) colistin-resistant isolates in six patients. A time and space cluster for five patients was found. Epidemiology typing showed that two isolates belonged to clone A (pts. 1, 5) and the rest to clone B (pts. 2–4) with 69% similarity. Clone A isolates were coproducers of *bla*NDM-like and *bla*OXA-48-like and had *mgrB*-mediated colistin resistance (40%). Clone B isolates had only *bla*OXA-48-like and intact *mgrB* genes. All isolates were negative for *mcr-1*, *-2*, *-3*, *-4*, and *-5* genes. *Conclusions:* The study describes a within-hospital spread of two clones of COLr CR-Kp with a 60% mortality rate. Clone A isolates were coproducers of NDM-like and OXA-48-like enzymes and had *mgrB*-mediated colistin resistance. Clone B isolates had only OXA-48-like enzymes and intact *mgrB* genes. No plasmid-mediated resistance was found. The extremely high mortality rate and limited treatment options warrant strict measures to prevent outbreaks.

## 1. Introduction

HAIs (hospital-acquired infections) account for 5–15% of all admissions worldwide (9 million), but the rate is probably higher because of significant underreporting [1]. The total annual cost for the five significant HAIs in the USA is USD 9.8 billion [2]. Almost 100 years after the discovery of antibiotics, we are faced with unprecedented antibiotic resistance, leading to a catastrophic crisis worldwide. Multidrug resistance (MDR) is defined as resistance to one or more antimicrobials from at least three different antimicrobial classes; extensive drug resistance (XDR) is non-susceptibility to at least one agent in all but two or fewer antimicrobial categories (i.e., bacterial isolates remain susceptible to only one or two categories); and pan-drug resistance (PDR) is resistance to all agents in all antimicrobial categories [3]. MDR pathogens account for 670,000 infections and 33,000 deaths in the European Union with healthcare costs of USD 1.1 billion [4,5]. Antimicrobial Resistance Collaborators estimated that 1.27 million deaths were attributable to antibiotic resistance during 2019. Six of twenty-three analyzed pathogens (*Escherichia coli*, *Staphylococcus aureus*, *Klebsiella pneumoniae*, *Streptococcus pneumoniae*, *Acinetobacter baumannii*, and *Pseudomonas aeruginosa*) were responsible for 929,000 of these deaths [6]. A UK report warned that by 2050 approximately 10 million deaths would occur if no action was taken [7]. These figures, however, were questioned by others, mainly due to a lack of reliable estimates of the antibiotic resistance burden [8]. Nevertheless, WHO declared priority status to the most frequently reported MDR bacteria called “ESKAPE” (*Enterococcus faecium*, *Staphylococcus aureus*, *Klebsiella pneumoniae*, *Acinetobacter baumannii*, *Pseudomonas aeruginosa*, and *Enterobacter* species) due to their ability to escape the antibiotics [9,10]. The MDR Enterobacteriaceae are essential because they are part of the human microbiota. Among these, *K. pneumoniae* appeared to be a significant problem due to its ability to accumulate antibiotic resistance genes (ARGs) by de novo mutations or via horizontal gene transfer by plasmid transfer (conjugation) or by bacteriophage (transduction); thus, Navon-Venezia et al. called it a “source and shuttle for antibiotic resistance” [11]. Via the above-mentioned mechanisms, ARGs could be easily transferred from harmless commensals to pathogenic bacteria [12]. According to a recent survey, *K. pneumoniae* is among the five deadliest bacteria, with more than 500,000 deaths yearly [13]. The carbapenem-resistant *K. pneumoniae* was first reported in 2001 in the USA [14]. The main reason for the appearance of carbapenem-resistant isolates is the production of carbapenemases. They are classified into two main groups: the first is the serine active group (class A Klebsiella-producing carbapenemase (KPC) and class D oxacillinases (OXA; OXA-48 and OXA-181), and their variants, are the main representatives) and the second comprises class B metallo-carbapenemases (New Delhi Metallo beta-lactamase (NDM), Verona Integron metallo-carbapenemase (VIM), and Imipenemase (IMP) enzymes) [15]. The European survey of carbapenemase-producing Enterobacteriaceae (EuSCAPE) demonstrated that the epidemic of carbapenem-resistant *Klebsiella pneumoniae* in Europe is driven by “within-hospital transmission and interhospital spread rather than between countries” [16]. According to ECDC (2021), the MDR strains account for 21%, with an increasing trend for carbapenemase-producing strains (11.7%) [5].

The Balkans are considered a reservoir of MDR and XDR *K. pneumoniae*. NDM-1-producing strains appear to spread within a similar time frame (2013–2016) [4,17,18,19]. The first polyclonal outbreak caused by NDM-1-producing *K. pneumoniae* in Bulgaria was described in 2015 at our and other institutions [17,18]. In 2019, Markovska et al. demonstrated the rapid interregional spread of NDM-1-producing ST11 strains with plasmid-mediated carbapenem resistance [20]. Unfortunately, the last link of the chain was the emergence of colistin-resistant strains. Polymyxin was discovered in 1947 and has been available on the market since 1959 for the treatment of Gram-negative infections [21]. Due to its nephrotoxicity, in the 1970s it was replaced by other antibiotics. Due to the emerging MDR crisis, colistin was “re-discovered” in 2000 and began to play a strategic role in the treatment of MDR Gram-negative infections [22,23]. At the same time, the rapid increase in its use rapidly led to an increase in resistance [21]. The studies published in the period 2008–2011 demonstrated a rate of colistin-resistant *K. pneumoniae* (COLr CR-Kp) between 1.5% and 28% [21]. According to Binsker et al., citing the ATLAS database, the global colistin resistance rate for 2014–2019 varied between 2.6% and 4.6%, and between 2.4 and 3.4% for Europe [24]. The first cases in Bulgaria were reported by Markovska et al. in 2015, and in our institution and other hospitals in Sofia and other cities in 2016 [25,26,27,28]. After 2013, alarming colistin resistance among carbapenem-resistant *K. pneumoniae* was reported in the Balkans–Bulgaria (37%), Greece (40%), Romania (27.5%), Serbia (10.6%), Türkiye (25.5%), and Italy (27%) [24,26,27,28,29,30,31,32]. More recent work from Poland for the 2019/2021 period reported a resistance rate of 14.5% [33]. Unfortunately, there are not enough data for the COVID-19 and post-COVID-19 periods for our region. The imminent disaster highlights the need for emergent measures to prevent its spread and to find new treatments because of the high mortality rate (41–70%) [34,35].

This study aims to perform an epidemiological, clinical, and genetic analysis of a local outbreak of COLr CR-Kp.

## 2. Material and Methods

### 2.1. Bacterial Isolates and Patients

The colistin-resistant and carbapenem-resistant *K. pneumoniae* isolates observed among patients in the ICU unit of Military Medical Academy, Sofia from 1 January to 31 October 2023 were included in the study. The hospital has 800 beds and more than 40,000 admissions per year.

The identifications of the microbial isolates were performed by MALDI-TOF mass spectrometry (MALDI-TOF MS, Bruker Corp. Billerica, MA, USA), following the manufacturer’s instructions. 

### 2.2. Antimicrobial Susceptibility Testing

The antimicrobial susceptibility testing (AST) was determined by Vitek 2 (bioMerieux, Marcy-l’Étoile, France). The reference method broth microdilution (ComASP Colistin, Liofilchem, Roseto degli Abruzzi (Te) Italy) was used to perform AST of colistin. The results were analyzed according to the criteria of the European Committee on Antimicrobial Susceptibility Testing (EUCAST) [36].

### 2.3. Molecular-Genetic Investigations

All isolates were PCR screened for the presence of *bla*VIM, *bla*IMP, *bla*KPC, *bla*NDM, and *bla*OXA-48, as previously described [37]. The *mgrB* gene was amplified and sequenced with primers reported previously by Kanateli. Nucleotide and deduced amino acid sequences were analyzed and multiple alignments were performed using Chromas Lite 2.01 (Technelysium Pty Ltd., Brisbane, Australia) and DNAMAN version 8.0 Software (Lynnon BioSoft, Vaudreuil-Dorion, QC, Canada).

Total bacterial DNA was prepared using the boiling method. ERIC (Enterobacterial Repetitive Intergenic Consensus) PCR with ERIC1R and ERIC2 primer sets was performed as previously described [20]. Genetic similarity of ERIC fingerprints was determined using the simple clustering method, UPGMA (unweighted pair group method with arithmetic mean), and the Dice coefficient as a similarity measure (http://genomes.urv.cat/UPGMA/, accessed on 20 January 2024). A clone was defined if the isolate showed a coefficient of similarity above 0.8.

Plasmid-mediated colistin resistance determinants (*mcr-1*, *mcr-2*, *mcr-3*, *mcr-4*, and *mcr-5*) were investigated with multiplex PCR, as suggested by Lescat et al. [38]. The chromosomal *mgrB* gene was amplified with primer sets as described previously [39].

## 3. Results

### 3.1. Bacterial Isolates and Patient’s Characteristics

A total of 379 MDR-*K. pneumoniae* isolates were isolated in the ICU unit, of which 333 (87.9%) were carbapenem resistant. Of these, six patients had nine (2.7%) colistin-resistant isolates. A time and space cluster was observed for five patients with eight colistin-resistant *K. pneumoniae* isolates. They were treated in the same room in the ICU within a two-week overlapping interval. All COLr CR-Kp were isolated within 13 days (31.08–13.09). The characteristics of the patients and isolates are shown in Table 1 and Table 2. All patients, except the fifth, had previous endoscopic interventions and initially colistin-susceptible *K. pneumoniae*; however, in the table they are given as only COLr CR-Kp.

The putative index patient in the series was a 69-year-old woman who underwent a left nephrectomy due to acute bleeding after retrograde intrarenal surgery, lithotripsy, and JJ stent. The urine culture revealed colistin-susceptible, carbapenem-resistant *K. pneumoniae* (22.08, data not shown)*,* which was treated with a suboptimal dose regimen of colistin (2 × 1 MM U) due to kidney failure. The first COLr CR-Kp was isolated from the wound tissue (31.08). The wound was treated locally, but after seven days a reoperation was performed due to organ space infection and severe necrotizing fasciitis. The patient developed sepsis with a positive blood culture (8.09) caused by the same strain and died on the 42nd postoperative day (POD).

The second and third patients underwent a Traverso–Longmire procedure for pancreatic cancer; both had bile duct stenting one month before the operation. The first isolates from the bile during the index operation in both patients were colistin-susceptible, carbapenem-resistant *K. pneumoniae*. In the second patient, COLr CR-Kp was recovered from the wound (3.09) and ascites (12.09). In the third patient, it was also isolated from the wound (12.09). Both patients were treated in one surgical clinic and both underwent reoperation on the same day (12.09).

In the fourth patient, colistin-susceptible, carbapenem-resistant *K. pneumoniae* was found in the urine (31.09), which was also treated with a suboptimal dose regimen of colistin (2 × 1 MM U). COLr CR-Kp was isolated from the tracheobronchial tree (4.09), on the seventh POD followed by positive blood culture (13.09). In the fifth patient, COLr CR-Kp was isolated from the urine on the sixth POD (11.09). Three of the five patients died (60%).

### 3.2. Antimicrobial Susceptibility Testing

All investigated *K pneumoniae* isolates were PDR and showed identical results and were resistant to amoxicillin, ampicillin, ampicillin/sulbactam, amoxicillin/clavulanic acid, piperacillin/tazobactam, cefalexine, cefuroxime, cefixime, ceftazidime, ceftriaxone, cefepime, imipenem, meropenem, colistin, gentamicin, amikacin, levofloxacin, fosfomycin, ciprofloxacin, moxifloxacin, and trimethoprim/sulfamethoxazole.

### 3.3. Molecular-Genetic Investigations

PCR reactions confirmed the production of carbapenemases (NDM or/and OXA-48) in all eight isolates. Two clones were confirmed by ERIC PCR and UPGMA analysis showing a 0.69 coefficient of similarity. Clone A was a coproducer of *bla_NDM-like_* and *bla_OXA-48-like_* enzymes (isolates *K pneumoniae* 1, 2, and 8 (patients 1 and 5)), whereas clone B (isolates 3, 4, 5, 6, 7 (pts. 2–4)) harbored only *bla*_OXA-48-like_ enzymes. The entire *mgrB* gene was amplified by PCR. In the isolates from clone A, the *mgrB* genes could not be amplified, showing truncated genes. In clone B, there were amplicons in all three isolates (intact *mgrB* genes), (Table 1). All isolates were negative for *mcr-1, -2, -3, -4*, and *-5* genes.

A broad hospital infection-control campaign was initiated encompassing the other patients, staff, and working environment, but no other COLr CR-Kp strains were isolated. The campaign included tightened control on the patients with XDR infections, with isolation in boxes or single rooms with access only for dedicated personnel. The patients were reoperated on in a dedicated “septic” operative room without interference with the “clean” patients. Regular microbiological specimens were taken from all the personnel, clinics, and operating theatres at random. For acutely ill patients referred from other hospitals, a microbiological survey was performed at admission. The Antimicrobial Stewardship Program was continued with a more restrictive antibiotic policy. The antimicrobial treatment of the complex cases was discussed by a multidisciplinary team including microbiologists.

## 4. Discussion

The present analysis revealed two clones of COLr CR-Kp that coincided with the time and space in the ICU unit. The most important was clone A, co-producer of OXA-48-like and NDM-like enzymes, and harbored disrupted *mgrB* genes responsible for the colistin resistance. The isolates of this clone were resistant to all tested antimicrobials, leaving no therapeutic alternative. Clone B harbored only OXA-48-like and lacked mutations in *mgrB* genes, so the colistin resistance might be explained by other chromosomal mutations [38,39]. The finding suggests that our series represents a within-hospital spread of COLr CR-Kp with two foci that coincided in the time and space (ICU). We speculate that the second focus originates from the second and third patients, who were operated on and managed in one clinic by the same team with subsequent stays in the ICU close to the fourth patient.

Of note, all patients in the presented series, except the fifth, underwent endoscopic intervention before the index operation (two endourology procedures and two bile duct stentings). All of them had an initial culture of colistin-susceptible *K. pneumoniae*, which also demonstrates the within-hospital spread.

Our finding is similar to the EuSCAPE survey, which demonstrated the central role of the inter-hospital, but more pronounced, transmission at the hospital level, similar to the results of Markovska et al. [16,20]. Other authors, however, reported that “carbapenem resistance reveals remarkable diversity and unexplained mechanisms” and that not all outbreaks could be linked to transmissions [40].

The first and fourth patients were treated with suboptimal doses of colistin (2 × 1 MM U daily for ten days). In the first patient, COLr CR-KP was detected on the 9th day of the treatment with colistin, whereas it was detected in the fourth patient on the 5th day. However, we can only speculate that this selective antibiotic pressure might explain the transition from colistin susceptibility to COLr CR-Kp in our series [41].

Although it was declared as strategic by WHO, colistin has been increasingly used in clinical practice, with a steep increase after 2005 [20]. Moreover, in 2017, the overall consumption of polymyxins in food-producing animals in 28 EU countries was 340 times higher than that in human medicine [25]. An increasing trend was also found in our institution. The consumption of colistin increased from 0.5 definitive daily doses per bed-day in 2017 to 1.98 in 2022 for the whole hospital, and from 11.7 to 25.5 in the ICU. A logical consequence of this worrisome trend is the rapid emergence of colistin-resistant strains. A recent meta-analysis demonstrated a significant increase in bloodstream COLr CR-Kp during the last decade, from 3% in 2015 to 13% in 2020 and after [42]. In the Balkans, the first COLr CR-Kp strains were isolated in 2012, with rapid expansion leading to multiple outbreaks in Greece, Bulgaria, and probably other countries (Table 3) [28,29,30,31,32,43].

A recent study showed significant heterogeneity in the molecular mechanisms behind colistin resistance [44]. Colistin resistance in *K. pneumoniae* is driven by the change (decrease) in the negatively charged lipopolysaccharides on the membrane surface, which precludes the binding with the cationic colistin. Several genes, mainly encoding the proteins of two-component regulatory systems PhoPQ, PmrAB, and CrrAB, are responsible for the decrease in this negative charge and resistance [44,45]. PhoPQ and PmrAB are negatively regulated by the mgrB protein on the inner surface of the membrane. The recently described CrrAB two-component system also regulates the PmrAB system. Kim et al. demonstrated higher survival rates in *crrAB*-positive isolates with early exposure to high colistin concentrations [46].

In a recent meta-analysis, Yusof et al. reported a pooled prevalence of mutated colistin resistance in *K. pneumoniae* of about 75% [47]. The most common genetic mechanism of resistance includes mutations in the genes *mgrB* (88%), *pmrA/pmrB* (54%)*, phoQ* (44%), and *phoP* (36%). Plasmid-mediated resistance via *mcr-1* was noted in 14%, while other genetic mechanisms were noted in 40%. In Bulgaria, a recent study by Markovska et al. found a lack, disruption, or mutation of the *mgrB* gene in 9/37 cases (24%), whereas, in the rest, the mechanism of resistance was not elucidated [28]. No plasmid-mediated resistance was found. These data are lower than our results (lack of *mgrB* in three of eight colistin-resistant isolates (37.5%)). These genes, however, could not explain all cases with colistin resistance. Macesic et al. demonstrated multiple genetic events in 71% of clusters with more than two patients [46]. According to the authors, several other genes constitute the so-called secondary resistome. Moreover, they state that polymixin exposure with de novo mutations rather than transmissions lies behind the colistin resistance.

The effect of *mgrB* gene inactivation, however, goes beyond the colistin resistance. In a murine model, Bray et al. demonstrated that the inactivation of *mgrB* leads to increased environmental survival of *K. pneumoniae* and facilitates host-to-host transmission [48]. Given its ability to survive on different surfaces, these consequences make the mutations in *mgrB* of particular importance for the spreading of *K. pneumoniae* even after strict infection control measures in hospital settings.

A very important but poorly estimated phenomenon is the mgrB-induced heteroresistance to colistin. A recent work of Alousi et al. underscores that the colistin-resistant subpopulations in the background of selective colistin pressure may become dominant, with worrisome consequences. In their study, they found heteroresistance in 21.9% of the CR-*K. pneumoniae* isolates [49]. A recent series from Bulgaria reported 8% heteroresistance [28]. The heteroresistance may hamper the interpretation of the antibiogram and, if unrecognized, it may lead to selective colistin pressure. Therefore, the early detection of heteroresistance is crucial to avoid this phenomenon.

The mortality rate of the present series is 60% and 100% in cases with bloodstream infection, which is in unison with the literature [34,36,50]. As of today, a few treatment options exist, such as ceftazidime/avibactam with or without aztreonam, plazomicin (not approved by EMA), cefiderocol, and fosfomycin. If the MIC of imipenem is below 8 mg/L, it could also be included in combination schemes. An excellent review by Petrosillo et al. demonstrated the characteristics of these antimicrobials and highlighted the need for an analysis of the meropenem MIC value, and OXA-48-like and NDM status, to guide the treatment [51].

The extremely high mortality rate and limited treatment options warrant strict measures to prevent outbreaks. Given the overtaking bacterial resistance and the difficult control of the chaotic and frequently defensive use of antibiotics, it appears more prudent to improve the prevention. Despite the high risk of bias (99%), the published literature suggests a sustained potential for reduction in HAI rates of between 35% and 55% using multifaceted interventions irrespective of a country’s income level [52]. Keeping the ten golden rules for optimal antibiotic use is of paramount importance [53].

The main limitation of the present study is the small sample size, which might be overcome with a multicenter national survey. Future research should be focused on a better understanding of the role of the number of the genes involved in colistin resistance, the role of CrrAB, phoPQ, and PmrAB, the early detection of heteroresistance, the influence of the dosage of colistin, and the duration of the treatment with this resistance [44,46]. Last, but not least, solving the puzzle also requires better elucidation of the biological cost of the gene mutations (increased environmental survival and facilitated host-to-host transmission) [48].

## 5. Conclusions

The present study describes a within-hospital spread of two clones of COLr CR-Kp with a 60% mortality rate. Clone A comprised co-producers of NDM-like and OXA-48-like enzymes and had mgrB-mediated colistin resistance. Clone B isolates had only OXA-48-like enzymes and intact mgrB genes. No plasmid-mediated resistance was found. The study also confirms the central role of the transmission at the hospital level, not only for COLr CR-Kp, but also for colistin-susceptible *K. pneumoniae*. The extremely high mortality rate and limited treatment options warrant strict measures to prevent the outbreaks. The decisive first step is to increase awareness about this threat and to implement in practice well-known principles, such as a hospital surveillance system, prevention, closer collaboration with the microbiology laboratory, an Antimicrobial Stewardship Program with a restrictive antibiotic policy, and creation of a multidisciplinary team discussing the antimicrobial treatment of the complex cases.

## Figures and Tables

**Table 1:** Characteristics, treatments, and outcomes of the included patients.

Patients	Gender	Age	Diagnosis	Intervention	Previous Intervention	ICU Stay	Outcome	Treatment
1	f	69	Acute kidney bleeding	Left nephrectomy	* RIRS + LT + JJ stent cystoscopy	18.08–3.10	died	amp/sulb, ceftriaxon, meropenem, colistin
2	m	71	Pancreatic cancer	Traverso-Longmire	bile duct stent	1–5.09 12.09–2.10	died	piperacilin/tazobactam, cfp/sulb, linezolid, colistin
3	m	46	Pancreatic cancer	Traverso-Longmire	bile duct stent	18–19.08 12–14.09	discharged	piperacilin/tazobactam, ciprofloxacin, colistin
4	m	82	Urine bladder cancer	Cystectomy	-	28.08–14.09	died	amp/sulb, levofloxacin, doxycycline, colisitn
5	m	56	Perforated duodenal ulcer	Suture	-	5–15.09	discharged	meropenem

Abbreviations: * RIRS—retrograde intrarenal surgery; LT—lithotripsy; amp/sulb—ampicillin/sulbactam; cfp/sulb—cefoperazone/sulbactam.

**Table 2:** Source and characteristics of the isolates.

Patient	Isolate	Sample	Date of Isolation	Carbapenemase	ERIC *	*mcr* 1–5	*mgrB*
1	1	wound	31.08	OXA-48-like and NDM-like	A	NEG	NEG
	2	blood culture	8.09	OXA-48-like and NDM-like	A	NEG	NEG
2	3	wound	3.09	OXA-48-like	B	NEG	POS
	4	ascites	12.09	OXA-48-like	B	NEG	POS
3	5	wound	12.09	OXA-48-like	B	NEG	POS
4	6	tracheo-bronchial	4.09	OXA-48-like	B	NEG	POS
	7	blood	13.09	OXA-48-like	B	NEG	POS
5	8	urine	11.09	OXA-48-like and NDM-like	A	NEG	NEG

Abbreviations: * ERIC—Enterobacterial Repetitive Intergenic Consensus, NEG—negative, POS—positive.

**Table 3:** The rate of colistin resistance in carbapenem-resistant *K. pneumoniae* in Balkan countries.

Author	Country, Study Period	% of MDR
Markovska, et al. [28]	Bulgaria, 2017–2018	37
Galani, et al. [30]	Greece, 2014–2016	40.4
epi-net.eu/records/12313/12313/[29]	Romania, 2018	27.5
Palmieri, et al. [32]	Serbia, 2013–2017	10.6
Cizmeci, et al. [31]	Türkiye, 2016	27.5

## Data Availability

The raw data supporting the conclusions of this article will be made available by the authors on request.
